# Resolving spatial inconsistencies in chromosome conformation measurements

**DOI:** 10.1186/1748-7188-8-8

**Published:** 2013-03-09

**Authors:** Geet Duggal, Rob Patro, Emre Sefer, Hao Wang, Darya Filippova, Samir Khuller, Carl Kingsford

**Affiliations:** 1 Lane Center for Computational Biology, School of Computer Science, Carnegie Mellon University, Pittsburgh, PA 15213, USA; 2 Department of Computer Science, University of Maryland, College Park MD 20742, USA

**Keywords:** 3C, Chromosome conformation capture, Metric violations, Triangle inequality, Graph embedding

## Abstract

**Background:**

Chromosome structure is closely related to its function and Chromosome Conformation Capture (3C) is a widely used technique for exploring spatial properties of chromosomes. 3C interaction frequencies are usually associated with spatial distances. However, the raw data from 3C experiments is an aggregation of interactions from many cells, and the spatial distances of any given interaction are uncertain.

**Results:**

We introduce a new method for filtering 3C interactions that selects subsets of interactions that obey metric constraints of various strictness. We demonstrate that, although the problem is computationally hard, near-optimal results are often attainable in practice using well-designed heuristics and approximation algorithms. Further, we show that, compared with a standard technique, this metric filtering approach leads to (a) subgraphs with higher statistical significance, (b) lower embedding error, (c) lower sensitivity to initial conditions of the embedding algorithm, and (d) structures with better agreement with light microscopy measurements. Our filtering scheme is applicable for a strict frequency-to-distance mapping and a more relaxed mapping from frequency to a range of distances.

**Conclusions:**

Our filtering method for 3C data considers both metric consistency and statistical confidence simultaneously resulting in lower-error embeddings that are biologically more plausible.

## Background

Chromosome conformation capture (3C) is an experimental technique designed to observe how the genome folds in a cell [1]. Measurements from 3C experiments have been used to construct three-dimensional models of chromosomes at a higher resolution than what is possible with light microscopy [2], and these models are correlated with long-range regulation [3], chromatin accessibility [4], as well as cancer-related genome alterations [5]. Since its introduction, the 3C technique has become widely adopted and has been applied to bacterial, yeast, fruit fly, and human genomes [3,6-13].

Measured interactions between genomic locations are aggregated into a chromosome conformation graph. The frequency of an interaction between a particular pair of genomic locations in the assayed population of cells can be converted to a distance, and this mapping allows the graph to be embedded in three dimensions. Before embedding, interactions in 3C graphs are usually filtered so that only interactions with unusually high frequencies given their genomic distance are kept. For example, contact matrices normalize the observed frequency of an interaction within a chromosome by the expected frequency within an entire genome (e.g. [4,14]) while others more explicitly model the distribution of interaction frequencies (e.g. [3,6]). In this sense, traditional statistical filtering methods retain high-confidence interactions.

However, because 3C measurements are aggregated over millions of cells, the distances associated with these high-confidence interactions are often metrically inconsistent. For example, among 2,257,241,015 triplets of measurements that form triangles in the yeast 3C data of Duan et al. [6], 679,480,886 (30%) do not satisfy the triangle inequality. These inconsistencies make it difficult to reason about conformational properties of the genome. Further, existing filtering procedures do not use relationships between the edges to, for example, discard high-confidence edges that are apparently inconsistent with many lower-confidence edges, or to include seemingly low-confidence edges that are nonetheless consistent with many others.

To address these shortcomings, we introduce the idea of metric filtering where we seek a high-confidence metrically consistent subset of the 3C graph. We frame the procedure as a family of optimization problems where we want to find a subgraph of high total weight (confidence) such that the set of chosen edges satisfies metric constraints of various stringency. We show that this family of problems, like embeddability in $R^{3}$[15], is NP-hard, and provide four algorithms for the approximate solution of the least and most stringent versions.

We apply the metric filtering algorithms to 4C measurements for budding yeast [6] and show that the heuristics are able to find near-optimal solutions to the variant of the problem where only triangles are consistent. Despite the additional metric constraints, the selected set of edges is often of higher total confidence than the data set considered in Duan et al. [6] that had an estimated 1% false discovery rate (FDR).

We show that embeddings based on these filterings have lower embedding error than those based on an existing filtering technique [6]. The structures also exhibit lower variation when different initial conditions are chosen for a previously proposed non-linear optimization embedding technique. Finally, we provide anecdotal evidence that the structure resulting from the metrically filtered interactions is in better agreement with known biology than the structure derived using standard filtering techniques. The improved agreement is a result of the metric filtering being able to include longer-distance, but lower-confidence, interactions.

## Problem definition

### Problem 1 (Consistent-k-Paths)

Given an integer *k*≥2, and a graph *G*=(*V*,*E*), where each edge *e*∈*E* is associated with a non-negative length *d*(*e*) and a positive reward *r*(*e*), find a subset *S*⊆*E* of edges that maximizes $R(S)=\sum_{e\in S}r(e)$ and such that, for all *e*∈*S* and for any path $P_{e}^{k}$ in *G* of *k* or fewer edges in *S* joining the endpoints of *e*, the following condition holds:

(1) $$\underset{e^{\prime }\in P_{e}^{k}}{\sum }d(e^{\prime })\geq d(e).$$

In other words, we seek the highest *total reward* subgraph where the length of every chosen edge is shorter than any path of *k* or fewer hops joining the endpoints of that edge. If an edge satisfies condition (1) for a given *k*, we say it is *k*-*consistent*, or simply *consistent* if *k* is clear from the context. If the edge is not consistent, it is *violated*.

Consistent-k-Paths is a family of problems parameterized by *k*. The value of *k* allows the strictness of the metric condition to be varied. To obtain an idea as to how relatively stringent the filterings are, we focus on the two extreme cases of *k*=2 and *k*=|*V*|−1. The strictest condition is *k*=|*V*|−1, when every alternative path must be at least as long as the direct edge connecting the endpoints of the path, while the most lenient condition is *k*=2, where consistency is only enforced for triangles. Because of their importance, we give names to these two special cases.

### Definition 1 (ConsT)

ConsT is an instance of Consistent-k-Paths with *k*=2, i.e. every triangle must satisfy inequality (1).

### Definition 2 (ConsP)

ConsP is an instance of Consistent-k-Paths with *k*=|*V*|−1 implying that all paths are consistent.

The ConsT formulation (and any formulation with *k*<|*V*|−1) is motivated by the fact that each measured distance is associated with some uncertainty that propagates when summing distances over longer paths. The ConsP property is more strict, requiring all paths to be consistent, but it suffers from the propogation of errors as distances are summed over large paths.

## NP-Hardness of Consistent-k-Paths

### Theorem 1

Consistent-k-Paths is NP-hard for *k*>1.

### Proof

Reduction from Independent Set: given $\ell \in Z_{\geq 0}$ and a graph *G*=(*V*,*E*), construct a graph *H*=(*V*∪{*u*},*E*∪*E*^′^) as follows. Let *d*(*e*)=*r*(*e*)=3 for all *e*∈*E*. Create a new vertex *u*, and a new set of edges *E*^′^={{*u*,*v*}∣*v*∈*V*}. Set *d*(*e*)=*r*(*e*)=1 for all *e*∈*E*’. We show that *G* has an independent set of size ≥*ℓ* ⇔ *H* has a solution of total reward *R*≥3|*E*|+*ℓ*. Note that a violating path in *H* contains exactly 2 edges and, along with the violated edge, forms a triangle. This is because every edge in *H* has *d*≥1, so that every path containing 3 or more edges will have a total *d*≥3. Such a path is as long as any edge in *H*, and hence can violate no edge. It follows that this reduction applies for all *k*≥2.

⇒ Let *S*⊆*V* be an independent set of size ≥*ℓ*. Choose all edges of *E* and the edges *E*_*S*_={{*u*,*w*}∣*w*∈*S*}. The total reward of this set is *R*=3|*E*|+|*S*|≥3|*E*|+*ℓ*. Since all of the edges in *E* have *d*=3, all 2-hop paths formed by these edges have *d*=6 and do not violate eq. (1). Further, since *S* is an independent set, no triangle involving *u* is selected. Therefore, the graph induced by the selected set of edges, *E*∪*E*_*S*_, is consistent.

⇐= Assume *E*^∗^ is a solution to the Consistent-k-Paths problem with *R*≥3|*E*|+*ℓ*. First, note that no triangle {*u*,*w*},{*w*,*v*},{*v*,*u*} can be selected since this would violate {*v*,*w*} because *d*(*v*,*u*)+*d*(*u*,*w*)<*d*(*v*,*w*). Due to the following argument, we assume, without loss of generality, that all edges of *G* are chosen: Suppose a pair of edges {*u,v*} and {*u,w*} was chosen and edge {*v,w*} exists in *E* but was not chosen. Then we can remove {*u,v*} and add {*v,w*}. This is still a solution with reward ≥3|*E*|+*ℓ*, since the swap only increases the value of the solution. Repeated application of this will produce a solution of cost ≥3|*E*|+*ℓ* that includes all of *E*.

In the transformed solution, the edges of *E* contribute a reward of 3|*E*|. Further, to avoid violating edges, the endpoints of selected edges adjacent to *u* must form an independent set, and to achieve a total reward *R*≥3|*E*|+*ℓ*, there must have been an independent set size ≥*ℓ*. □

### Corollary 1

Consistent-k-Paths restricted to *r*(*e*)=1 for all *e*∈*E* is NP-hard for *k*>1.

### Proof

Apply the same reduction as above, except that *r*(*e*)=1 instead of 3 for all *e*∈*E*, and using a total reward threshold of *E*^∗^≥|*E*|+*ℓ* instead of 3|*E*|+*ℓ*. In the new formulation, *E*^∗^ might include two edges {*u*,*w*} and {*u*,*v*} without picking {*v*,*w*}∈*E* if it exists. However, any such solution can be transformed into one of the form above with equal cost by removing {*u*,*w*} and adding {*v*,*w*}. This neither changes the number of edges nor decreases the total reward of *E*^∗^, and the proof can proceed as in Theorem 1. □

## Algorithms

Since ConsT and ConsP are NP-hard, it is unlikely that there exist algorithms that solve these problems in polynomial time. Thus, we have developed several approximation algorithms and heuristics to tackle them in practice. We present five algorithms below; the first three apply to the ConsT problem while the latter two apply to the ConsP problem.

### A set-cover-based algorithm

We formulate ConsT as a *minimum weight* set cover problem by removing the lowest weight set of edges that restores consistency, and therefore maximizes the weight of the remaining graph (the complement of the original problem). Let $\bar{\Delta }$ be the set of violated triangles in *G* (where a triangle is *violated* if it does not obey the triangle inequality). For edge {*u*,*v*} in *E*, let *S*_*uv*_ be the subset of triangles in $\bar{\Delta }$ that contain {*u*,*v*}, and let *C*={*S*_*uv*_ for all {*u*,*v*}∈*E*}. Define the cost *c*(*S*_*uv*_)=*r*({*u*,*v*}). We then seek the smallest weight collection of sets *S*_*uv*_ that cover all the violated triangles $\bar{\Delta }$, a direct application of minimum-weight set cover. Removing the edges corresponding to each chosen subset *S*_*uv*_ will resolve all of the violated triangles. This problem can be approximated using either an LP relaxation or a greedy algorithm [16]. Note that, since each violated triangle belongs to at most 3 sets of the collection *C*, there is an algorithm that finds a solution to this Set-Cover instance with a cost no more than 3 times OPT[16]. For the experiments described here, we use the greedy algorithm. There exist exact algorithms for the related hitting set problem [17], but these are only efficient when the number of edges that need to be removed is small, which is not what we observe in the 3C data we analyze.

### A hierarchical maximum cut approach

Another approach to solve ConsT uses a solution to the Max-Cut problem to find a maximum weight (i.e. maximum total reward) cut-set *E*’ separating vertices of *G* into *V*_1_ and *V*_2_. Because *E*’ is bipartite, it will have no triangles, and thus, no violated triangles. The Local-Cut algorithm [18] guarantees that *E*’ has at least 1/2 the total reward of *G*. Therefore, this algorithm is a 1/2-approximation to ConsT. We add all edges in *E*’ to the growing solution set *E*^∗^. Then, for every pair of edges {*u*,*v*},{*u*,*w*} in *E*’, if there is an edge {*v*,*w*}∈*E*∖*E*’ that forms a violated triangle, we remove {*v*,*w*} from *G*, and we recursively apply this procedure to the two partitions induced by the maximum cut. Because the subgraphs induced by *V*_1_ and *V*_2_ only contain the set of edges that form non-violating triangles with the edges in *E*’, the constructed solution contains no triangle violations.

### Integer linear program

The ConsT problem can be expressed succinctly as an integer linear program (ILP), mirroring a standard ILP for set cover:

(2) $$\begin{array}{l} \text{maximize}\;\;\underset{e\in E}{\sum }x_{e}r(e)\; \end{array}$$

(3) $$\begin{array}{lllll} \text{subj. to} & \;\;x_{e_{1}}+x_{e_{2}}+x_{e_{3}}\leq 2\; & \text{for}\;\{e_{1},e_{2},e_{3}\}\in \bar{\Delta } & \; & \;\\ & \;\;x_{e}\in \{0,1\}\; & \; \end{array}$$

where $\bar{\Delta }$ are the set of metrically inconsistent triangles in *G*. Of course, (2) is computationally difficult to solve. However, by relaxing the condition in eq. (3) to 0≤*x*_*e*_≤1, we obtain a linear program which can be solved efficiently and whose objective provides an upper bound on total confidence of the optimal metrically consistent solution.

### Taking the union of shortest paths

Let $P_{\text{uv}}$ be the set of edges in all shortest paths (according to *d*) going from node *u* to node *v* in *G*=(*V*,*E*). A feasible solution to ConsP is to take the edges in $\underset{\{u,v\}\in E}{⋃}P_{\text{uv}}$. We call this the SP-Union heuristic. The intuition behind it is that, by definition, no edge that is part of some shortest path in *G* can be violated. Assume such an edge {*u*,*v*} was violated. Then, there must exist some path *p* between *u* and *v* with *d*(*p*)<*d*(*u*,*v*). However, this contradicts the fact that {*u*,*v*} belongs to some shortest path, because we could replace {*u*,*v*} with *p* and shorten this path.

Unfortunately, there may be an exponential number of shortest paths in *G*. However, by removing from *E* all edges that are not part of some shortest path, we can obtain the desired set of edges without explicitly enumerating all shortest paths. The SP-Union heuristic first computes, for every edge {*u*,*v*}∈*E*, the length of the shortest path between its endpoints, *d*(sp_*u*,*v*_). Then, the solution is simply given by *E*^∗^=*E*∖{{*u*,*v*}∈*E*∣*d*(sp_*u*,*v*_)<*d*(*u*,*v*)}.

### Maximum spanning tree heuristic

The final heuristic, MST-Add, first constructs a maximum-reward spanning tree *T*=(*V*,*E*_*T*_) on *G*=(*V*,*E*) and adds its edges to *E*^∗^. This can be computed using any standard maximum-weight spanning tree algorithm. By construction, *T* has a high total reward. Since it is a tree, it contains no cycles, and hence no violations. We sort the remaining edges *E*∖*E*_*T*_ by their reward and, iterating through them in descending order, add them to *E*^∗^ if they do not violate any shortest paths in the growing *E*^∗^.

### Metric filtering with uncertain distances

In ConsT and ConsP, we assume that every observed frequency *f*(*e*) maps to a single spatial distance. However, it is more plausible that an observed frequency maps to a range of distances. In this case, we suppose that there is a range of distances [*l*(*e*),*u*(*e*)] where *l*(*e*) and *u*(*e*) represent the lower and upper bounds of all valid distances to which the frequency *f*(*e*) of edge *e* can map.

Given such a range for every edge *e*, the new condition for metric consistency (previously inequality (1)) becomes:

(4) $$\underset{e^{\prime }\in P_{e}^{k}}{\sum }u(e^{\prime })\geq l(e).$$

This allows distances assigned to the edges to be “flexible,” and they can be extended or contracted within the range’s bounds to satisfy the new metric consistency condition (4). An edge *e* is *violated* only if, for some path of *k* or fewer hops connecting the endpoints of *e*, the sum of the upper bounds of the distances for edges *e*’ in this path is less than the lower bound of the distance range for *e*.

All of our algorithms can be modified in a straighforward way to use this relaxed definition of metric consistency. Now, the Set-Cover algorithm need only cover triangles that are violated according to (4). In the Max-Cut algorithm, we need only remove edges from the left and right bipartition that form triangles involving cut edges that are violated under the new definition. When computing the set of shortest paths (SP-Union) and the maximum-reward spanning tree (MST-Add), the shortest path lengths are now computed using *u*(*e*^′^) and, to test whether any edge *e* is violated, these lengths are compared to *l*(*e*).

## Computational results

### Weights for 3C interactions in budding yeast

We use the measurements from Duan et al. [6], who used a 3C variant called 4C to assay interactions for the entire *S. cerevisiae* genome during interphase with two experiments based on the HindIII MseI and MspI restriction enzymes. In total, Duan et al. measured 4,097,539 interactions across 4,193 genome fragments (nodes). Each of these interactions *e* is associated with a frequency *f*(*e*) — the number of times it was observed.

Duan et al. process these raw frequency counts to derive several other measures for each interaction. A spatial distance *d*(*e*), which we use as the edge length in condition (1), is assigned to every interaction using a frequency-to-distance mapping based on the observed inverse relationship between genomic separation and frequency for intra-chromosomal interactions. Such a distance mapping is common to most approaches that seek embeddings of 3C data [3,6,10,19]. Because the distance mapping is based on intra-chromosomal interactions, we have more confidence in the spatial distances *d*(*e*) for interactions within a chromosome. Therefore, in most of the experiments below, we consider each chromosome individually. Table 1 gives the sizes of the graphs for each chromosome of yeast.

**Table 1:** Sizes of yeast chromosome conformation graphs

**Chr**	**| *V *|**	**| *E *|**
1	54	1046
2	311	31600
3	100	3738
4	521	86126
5	184	11243
6	92	3050
7	404	52224
8	192	13226
9	149	7738
10	257	2234
11	253	20232
12	361	38052
13	331	36792
14	283	26531
15	368	43807
16	333	37054

|*V*| is the number of interaction fragments per chromosome, and |*E*| is the number of intra-chromosomal edges.

Duan et al. also compute a p-value for every *e*. Using these p-values, they further derive a “q-value” that accounts for multiple hypothesis testing caused by the large number of edges sampled. See the “Computational methods” of the supplementary material of Duan et al. for a description of how the q-values are computed. We reproduced their p-value and q-value computations and use *r*(*e*)=1−*q*(*e*) as the reward for including an edge in the solution in condition (1). The value 1−*q*(*e*) is a measure of confidence: high values indicate low p-values, which indicate that interactions occur with a frequency that one would not expect by chance.

The input to the filtering procedures is thus the graph *G*=(*V*,*E*) where *V* is a set of restriction fragments and *E* is the set of interactions. The distance on an edge *e* is *d*(*e*) and the reward on an edge is the confidence *r*(*e*). The goal of metric filtering is to find a subgraph with high confidence (i.e. generally low p-values) with no metric violations as defined by condition (1).

### Ability of the algorithms to find high-weight subgraphs

**Figure 1:**
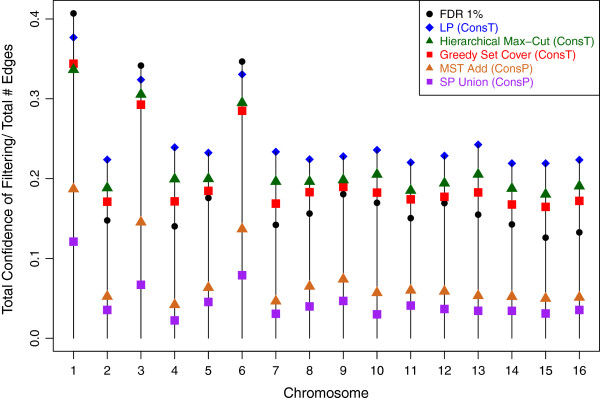
**Metric filtering performance on various chromosomes of yeast.** The total confidence that each algorithm recovers normalized by the total number of edges is plotted. Higher values are better. The objective value of the linear program (blue diamonds) gives an upper bound for both the ConsT and ConsP solutions. We also compare our algorithms to the Duan et al. filtering method at FDR 1% (black circles) which is their largest and highest-confidence filtered interaction set. (A bug in the SP-Union script in the conference version of this paper [20] erroneously led to plotting the sum of edge q-values instead of the sum of confidence. The plots have been updated to correctly plot total confidence with little qualitative difference).

The heuristics of the Algorithms section were tested on each of the 16 yeast chromosomes, and Figure 1 summarizes the algorithms’ ability to find high-confidence solutions. In 15 out of 16 cases, Max-Cut finds a ConsT subgraph with the largest total confidence (Figure 1, green triangles). However, in all 16 chromosomes, the Set-Cover method finds a graph of nearly the same quality, indicating that this method is competitive in terms of its ability to optimize the objective function.

The ConsT subgraphs have similar—and usually higher—total confidence than FDR 1% while eliminating all violated triangles (compare black circles with green triangles and red squares in Figure 1). The set of interactions at FDR at 100*q*^′^% is the set of edges originally considered by Duan et al. with *q*<*q*’. Both Set-Cover and Max-Cut achieve total confidence that is higher than the Duan et al. FDR 1% filtering for all but the smallest chromosomes (1, 3, and 6). Even in those cases, Set-Cover and Max-Cut solutions are no more than 25% away from the FDR 1% total confidence.

Due to the NP-hardness of the problems, optimal solutions for ConsT and ConsP are difficult to obtain. However, the ConsT problem can be expressed as an integer linear program (2). While this ILP is also difficult to solve, its linear relaxation is solvable in practice and provides a provable upper bound on the value of the optimal solution, shown as blue diamonds in Figure 1. This bound reveals that the Set-Cover and Max-Cut approaches find solutions that are close to optimal. Experiments on all chromosomes achieve total confidence values that are at least 70% of the linear program upper bound, and four cases achieve total confidence of around 90% of the upper bound. Since the LP overestimates the optimal value, it is likely that the heuristics provide solutions that are much closer than 70% of the true optimal solution.

The algorithms for ConsP (MST-Add and SP-Union) find graphs with far fewer edges and far lower total confidence than any of the solutions for ConsT (Figure 1), and they sacrifice a significant proportion of the total confidence to obtain a completely metric subgraph. This is a strong indication of how much more strict the ConsP condition is compared with ConsT. In addition, the SP-Union algorithm performed worse then MST-Add. The severe condition required by ConsP is likely too strict for the noisy 3C data, and ConsT provides a more reasonable trade-off between avoiding metric violations and keeping a useful fraction of the interactions.

### Metric filtering produces lower-error embeddings

The ConsT and ConsP filterings both result in lower-error embeddings than their associated confidence-ranked filterings when embedded using a nonlinear optimization technique. To control for the size of filterings we compare a metric filtering with *m* interactions to an associated set of the *m* highest confidence interactions (C-Rank). The embedding attempts to place nodes to minimize the sum-squared error of $\sum_{e\in E^{\prime }}{\left(o(e)-d(e)\right)}^{2}$ between the original *d*(*e*) and the embedded *o*(*e*) distance. The Set-Cover filtering of chromosome 1 resulted in a mean sum-squared error of 0.97 across 10 embeddings while C-Rank resulted in an error of 1.58. Similarly, MST-Add had an average error of 0.067 while C-Rank produced an error of 0.39. Our improved performance may be due to the fact that metric violations result in distance contradictions that cannot be resolved by the optimization procedure.

To confirm the hypothesis that metric violations cause increased errors in the embeddings, we systematically re-introduced violated triangles using the following procedure. We choose a triangle {*u*,*v*,*w*} at random. If *d*(*u*,*v*)<*d*(*u*,*w*), then we set *d*(*u*,*v*)=*α*|*d*(*v*,*w*)−*d*(*u*,*w*)|. Otherwise, we set *d*(*u*,*w*)=*α*|*d*(*v*,*w*)−*d*(*u*,*v*)|, for some choice of 0<*α*<1. As the percentage of violated triangles increased, the embedding error increased as well with 1.2,1.4,1.6,2.0,2.3,2.8 average error for 10,20,30,40,50,60% violated triangles respectively with *α*=0.9. Metric filtering therefore has the desirable property of removing the embedding error that results from the existence of violated triangles.

### Metric filtering produces low-variance, more biologically plausible embeddings

We embedded the various sets of filtered constraints for the chromosomes using an established non-linear optimization technique [6,9] that incorporates chromatin packing constraints consistent with known biology in yeast. We obtained ensembles of structures by providing random initial conditions for our implementation of this optimization, a technique previously used to study conformational differences between cancer and healthy genomes [3].

**Figure 2:**
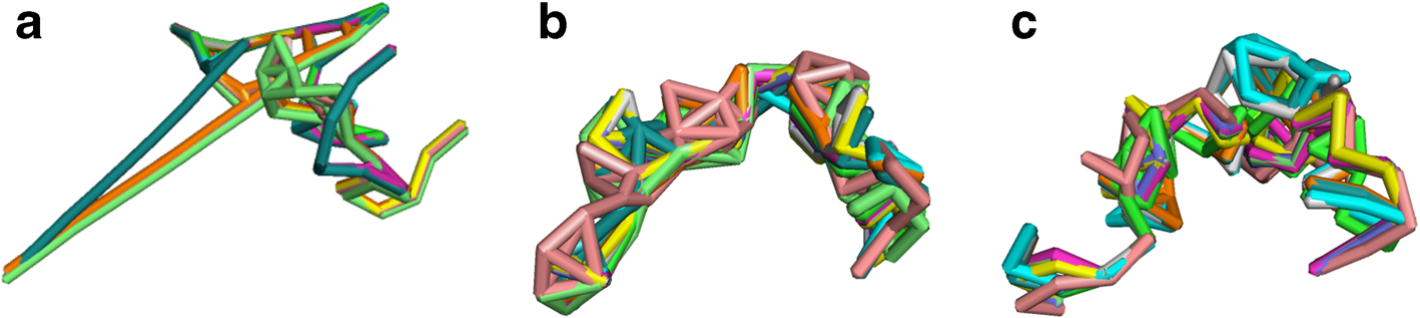
**Filtered graphs embedded in 3D.** Superposition of 10 embeddings for both ConsT and C-Rank filterings. **(a)**Set-Cover. **(b)**C-Rank of the same size as the Set-Cover. **(c)**Set-Cover after removing ≈20*%* of the lowest-confidence edges.

Ensembles of 10 embeddings for Set-Cover on chromosome 1 are shown in Figure 2a and the ensembles for a non-metric filtering with equal number of edges (obtained by taking the corresponding number of edges with the highest confidence) are in Figure 2b. We focus on observations for chromosome 1, but have observed similar trends for other chromosomes. For each filtering, the embeddings are aligned to each other using a maximum likelihood superpositioning technique [21].

#### Lower-variance embeddings

Both ConsT (Figure 2) and ConsP filterings produce ensembles of embeddings that are more homogeneous than those from the associated C-Rank sets as indicated by the superposition of structures in Figure 2. We can quantify the variance of an ensemble by computing the sum of the branch lengths of a minimum spanning tree of a complete graph where the nodes represent the embedded structures and the edge weights are the RMSD between the alignments of pairs of structures. The minimum spanning tree on this graph represents a parsimonious way to describe the variability among embedded structures. The MST-based variability between the Set-Cover and MST-Add embeddings of chromosome 1 are 0.17 and 0.0093 respectively while the MST variability of the associated C-Rank embeddings are much larger, at 0.26 and 0.32 respectively.

Low variance among the embeddings of metric subgraphs indicates that selecting edges for their metric consistency allows fewer highly different solutions to be found. This is desirable, because we do not want embeddings to be sensitive to the initial conditions of the optimization procedure. Further, because they were taken from a population of cells, the 3C measurements are in fact taken from an ensemble of structures. The large variance among C-Rank edges may reflect this fact. In contrast, the metric filtering appears to be selecting subsets of constraints that could plausibly represent a single structure. Hence, metric filtering may be one way to partially deconvolve the population-averaged measurements.

#### Biologically plausible embeddings

The ConsT embeddings result in telomere distances that match known microscopy distances better than the associated C-Rank set. A recent experiment [22] establishes that the distance between the telomeres of chromosome 1 in budding yeast are often about 1 *μ*m. The embeddings of C-Rank in Figure 2b have an average distance of 0.45 *μ*m while the embeddings of Set-Cover (Figure 2a) have an average distance of 0.96 *μ*m, which is a much better match to the experimentally observed value.

Despite having edge sets of the same or larger total confidence, the metric filtering produces very different structures than the C-Rank filtering. However, removing the 71 lowest-confidence edges from the ConsT embedding does result in a structure similar to the C-Rank filterings Figure 2c. Thus, it seems these lower-confidence, but metrically-consistent, interactions are crucial to obtaining the more distended structures that are more consistent with microscopy experiments.

### Analysis of the types of edges kept by metric filtering

**Figure 3:**
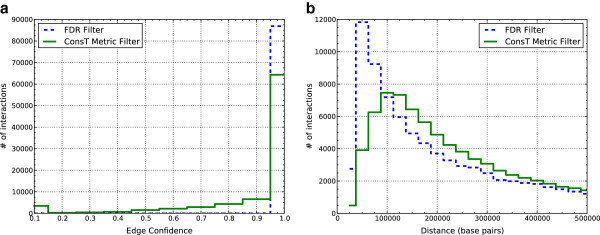
**Histogram of genomic distances and interaction confidences between C-Rank and ConsT.** Distances and confidences for both filtering methods were computed on the intra-chromosomal interactions. (**a**) The ConsT filtering kept more low-confidence edges than C-Rank (“FDR Filter” in the legend). (**b**) The ConsT filtering kept interactions with larger genomic distances than C-Rank..

For all chromosomes, both ConsP and ConsT keep more low-confidence edges than the C-Rank filtering (ConsT shown in Figure 3a). Although, in general, more low-confidence edges are kept, the ConsT filtering of chromosome 1 preserves overall higher distance interactions than the associated C-Rank filtering with a mean distance of 0.55 while the C-Rank filtering has a mean distance of 0.25 (all distances in *μ*m). Of these interactions, the high-confidence ones in the ConsT filtering (i.e. those above 0.8) have a mean distance of 0.31 while the C-Rank filtering has a mean distance of 0.25. This is due to the fact that the interactions in the C-Rank filtering are concentrated in a small region of chromosome 1, while the Set-Cover filtering distributes the interactions across the entire chromosome: for the interactions in the C-Rank set, but not in the ConsT filtering, 76 out of the 131 interactions lie between positions 75881 and 130646 of chromosome 1 while the densest region of similar size in the Set-Cover filtering has only 26 interactions. The preservation of larger-distance, higher-confidence interactions in the ConsT filtering is likely what results in the expanded structure where telomere distances are more in line with microscopy experiments. For the ConsP embedding, however, the large disparity in mean distance of interactions (C-Rank: 0.165, MST-Add: 1.69) is due mostly to low-confidence edges. This creates an undesirable structure that contains very little useful information about long-range interactions. This is another indication that the strictness of the ConsP filtering may be too severe compared with the more relaxed and biologically plausible ConsT approach. The inclusion of long-range interactions resulting from lower-confidence edges represent some of the most interesting and desired information obtained from 3C experiments. C-Rank necessarily ignores many of these long-range constraints, while the metric filtering allows the inclusion of both metrically consistent and higher-confidence constraints.

In addition, the ConsT method generally keeps interactions with larger genomic distances than C-Rank. The average genomic distance of C-Rank is 243.5 kilobases while the average genomic distance of Set-Cover is 285.0 kilobases (Figure 3b). Surprisingly, while ConsP keeps more low-frequency interactions, these tend to be at shorter genomic distances.

### Various heuristics result in very different sets of edges

**Figure 4:**
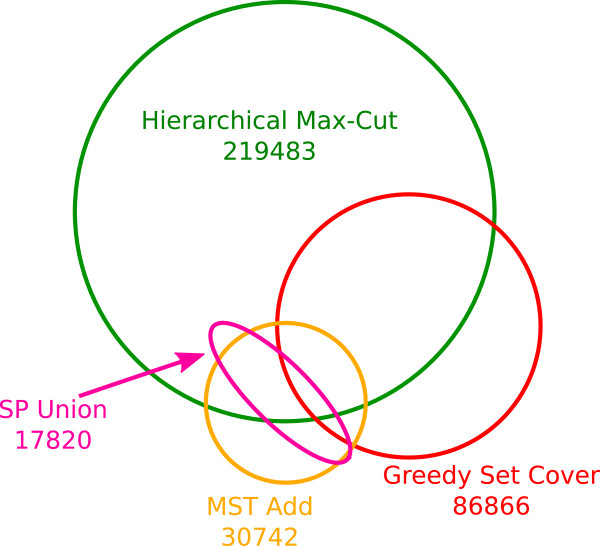
Intersections among metric edge sets.

Although the Max-Cut and Set-Cover algorithms aim to optimize the same objective and find subgraphs of approximately the same total weight, they result in very different edge sets (Figure 4). Further, their intersections with the most confident edges are also different: of the 219,483 edges returned by Max-Cut, 53% are among the top 219,483 most confident edges, while 60% of the 86,866 edges returned by Set-Cover are among the most confident edges (Table 2). The differing number of edges in solutions with similar total confidence also indicates that the edges in the Max-Cut solution are of lower average confidence.

**Table 2:** **Intersections with**C-Rank**sets of equal size**

**Algorithm**	**Inter. with**C-Rank
Set-Cover	51865
Max-Cut	116049
MST-Add	14210
SP-Union	7345

The structure of the graphs returned by Max-Cut is also very different than that of those returned by Set-Cover. The Max-Cut solution has very few triangles. For example, on chromosome 1 Max-Cut retains only 27 out of the original 10091 triangles while Set-Cover keeps 495. This difference is somewhat intuitive since Set-Cover is explicitly trying to throw away few triangles while Max-Cut is explicitly looking for triangle-free (i.e. bipartite) subgraphs. For fewer, higher-weight edges with many triangles the Set-Cover should be preferred. This is likely the scenario that is most applicable to 3C chromosome embedding. Because optimal solutions cannot be found for large instances, it is unclear at this point whether the large variation in the returned edge sets is due to the objective function admitting many solutions or whether, if optimal solutions could be found, they would all be similar.

The two algorithms designed for ConsP also result in very different graphs, but this is primarily because the MST-Add algorithm is far more successful at finding a good solution than the SP-Union approach. The two algorithms had similar intersections with the top-most confident edges: for MST-Add, 46% of the edges were among the top-most confident edges, while for SP-Union the fraction was 41%.

### Performance under uncertain distances

In the experiments conducted here, we set *l*(*e*)=(1−*ρ*)*d*(*e*) and *u*(*e*)=(1+*ρ*)*d*(*e*), 0≤*ρ*≤1. Here, *d*(*e*) denotes the distance corresponding to *f*(*e*) using the original frequency-to-distance mapping. The parameter *ρ* determines the size of the distance range to consider. When *ρ* is set to 0, no flexibility is allowed and condition (4) reduces to condition (1); when *ρ* is set to 1, the lower bound for the distance of an edge *e* goes to 0, allowing all edges to satisfy condition (4). Therefore, with *ρ*=1 no edges will be removed by the filtering. We systematically sample *ρ* in the range of 0 to 1 in increments of 0.01.

**Figure 5:**
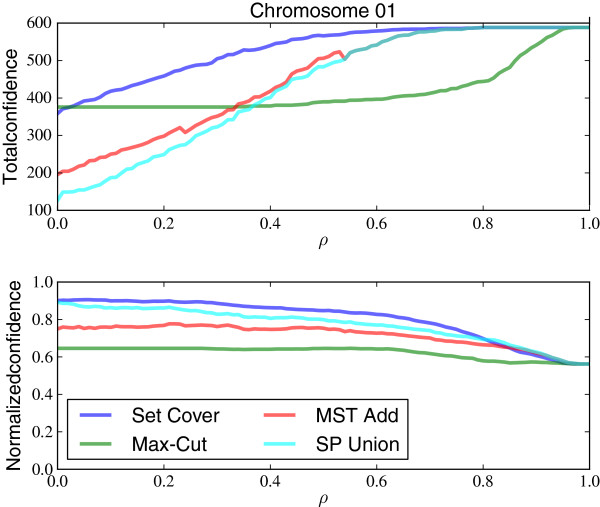
**Total and normalized surprises of included edges vs.*ρ*.** Total confidences (top) and normalized confidences (bottom) of included edges for different metric filtering algorithms on yeast chromosome 1 dataset. Normalized confidence is the total confidence divided by the number of edges in the output graph.

Using distance ranges *l*(*e*),*u*(*e*), we compute the total confidence included in the filtered graph as a function of the slack factor *ρ* (Figure 5). The total confidence for chromosome 1 gradually increases as a function of *ρ* and there is no *ρ* for which there is a sudden jump in the total confidence of the output graph. If some choice of *ρ* had lead to significantly more total confidence, filtering with ranges defined by that *ρ* would make sense if there was independent evidence that the computed distances are uncertain with that factor. Here, however, a small non-zero *ρ* does not substantially increase the confidence of the resulting graph, indicating that there are not many inconsistent edges that are on the cusp of metric consistency.

**Figure 6:**
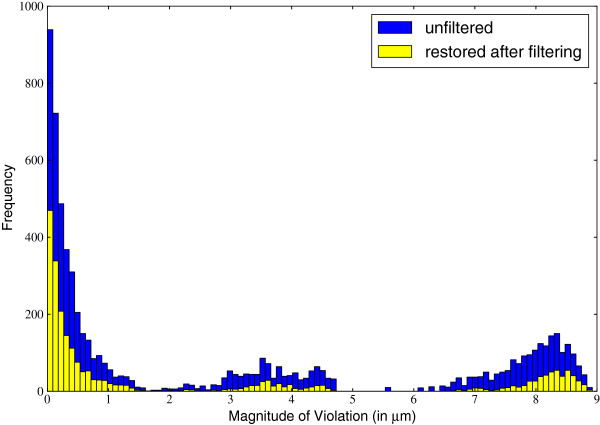
**Distribution of edge violation magnitudes.** Histogram of *d*(*u*,*w*)−[*d*(*u*,*v*)+*d*(*v*,*w*)] for all edges {*u*,*w*} in violating triangles in the original graph (total bar height) and the number of these edges that are included in the filtered graph when *ρ*=0 using the Set-Cover filtering (yellow portion). The blue portion of the bar represents the number of edges that are filtered out when *ρ*=0.

The linearly increasing total confidence observed in Figure 5 is largely explained by the distribution of the size of edge violations for edges included when *ρ*=0 (Figure 6). In any given error bin, a large fraction of the violated edges are corrected at *ρ*=0, providing more evidence that the algorithms generally perform well with a strict distance mapping. We also observe that the edges that are corrected by the metric filtering are not associated with any particular magnitude of error. For example, even though most of the violating edges are low error, they are not preferentially removed in the metric filtering. Because of this, the histogram of errors that remain to be resolved is more uniform in shape than the original distribution of errors, and therefore we would expect, as observed, a gradual increase in total confidence as *ρ* increases.

Between the ConsP algorithms, MST-Add obtains higher total confidences for different values of *ρ*, while SP-Union obtains higher average confidence on the edges in the output graph. In other words, SP-Union includes higher quality edges, and MST-Add includes more edges. A *ρ* of 0.34 is needed before MST-Add acheives the total confidence of Set-Cover with *ρ*=0. This provides more evidence that the ConsP condition is too strict.

Interestingly, although Max-Cut and Set-Cover perform similarly for low values of *ρ*, Set-Cover obtains the highest total (and normalized) confidence under most values of *ρ*. The Max-Cut method fails to include more edges when the metric consistency condition is relaxed because the cut it finds in the first pass is not affected by the choice of *ρ* and this cut includes most of the edges it will eventually keep. This resistance to the flexibility provided by (4) is another reason that Max-Cut is less favorable than Set-Cover.

### Practical running times of the algorithms

When applied to the largest yeast chromosome (chromosome 4), the Set-Cover and Max-Cut implementations take 21 seconds and 21.5 minutes to run respectively on an Opteron 8431 processor. The SP-Union and MST-Add methods take 2.25 minutes and <2 days respectively. The current implementation of MST-Add re-computes the shortest paths after every edge addition, and this could be substantially sped up with a dynamic shortest-paths method. The Set-Cover implementation is fast enough to be run on the entire yeast genome, including inter-chromosomal interactions, within 5 hours. In this case the ConsT filtering results in a significantly different edge set than the C-Rank embedding (the size of the intersection with C-Rank is only 350960 out of 657177 edges). The Set-Cover algorithm also yields a relatively high average confidence (0.88) when compared to the average confidence from C-Rank (0.97).

## Conclusions

We have provided evidence that a filtering scheme for 3C data that uses both statistical confidence and metric consistency as criteria produces sets of interactions that are more embeddable, and creates more consistent and more biologically plausible estimations for the 3D structures of the chromosomes. We show that such filtering in general is NP-hard, but by comparing to LP-based upper bounds, we empirically demonstrate that both a set cover approach and a hierarchical maximum cut algorithm produce nearly optimal solutions avoiding any violated triangles. Finally, we demonstrate that the methods can be extended in a straightforward way to account for ranges of allowable distances.

## Competing interests

The authors declare that they have no competing interests.

## Authors’ contributions

GD, RP, ES, HW and DF implemented the algorithms and performed the experiments. All authors helped design the algorithms and experiments, and helped write the manuscript. All authors read and approved the final manuscript.
